# Extracts of Vine Tea Improve Diet-Induced Non-Alcoholic Steatohepatitis Through AMPK-LXRα Signaling

**DOI:** 10.3389/fphar.2021.711763

**Published:** 2021-07-30

**Authors:** Yu-jun Chen, Hai-yan Song, Zi-wei Zhang, Qian Chen, Zhi-peng Tang, Ming Gu

**Affiliations:** Institute of Digestive Disease, Longhua Hospital, Shanghai University of Traditional Chinese Medicine, Shanghai, China

**Keywords:** adenosine monophosphate-activated protein kinase, liver X receptor α, non-alcoholic steatohepatitis, metabolic disorder, vine tea

## Abstract

Chinese vine tea can improve glucose and lipid metabolic disorders. However, its protective effects in non-alcoholic steatohepatitis (NASH) and its underlying molecular mechanisms remain unclear. Liver X receptor α (LXRα) inhibition and adenosine monophosphate-(AMP)-activated protein kinase (AMPK) activation can enhance control of NASH. AMPK activators have also been shown to inactivate LXRα. Here, the anti-NASH effects of vine tea extract (VTE) dosed at 1 g.100 g^−1^ diet were investigated using NASH mice challenged with a methionine and choline-deficient l-amino acid diet (MCDD) and a high-fat diet (HFD). Pharmacological mechanisms of VTE were explored using TUNEL staining, AMPK inhibition, Western blot, reporter assays, qRT-PCR analyses, and immunofluorescence. VTE treatment improved fatty liver in HFD-induced mice, while it alleviated the progression of NASH including protecting against liver lipid accumulation, steatosis, endoplasmic reticulum stress, apoptosis, inflammation, and functional injury in MCDD-fed mice. VTE reduced the action of hepatic lipogenic genes, F4/80, pro-inflammatory cytokines, CHOP, and cleaved Caspase-3 expression, while promoting expression of fatty acid oxidation genes CPT1α, *ß*. VTE also enhanced AMPK and blocked LXRα signaling in mouse livers. *In vitro* results indicated that VTE increased AMPK phosphorylation and reduced LXRα activity in HepG2 cells. Conversely, the antagonistic effect of VTE on LXRα was decreased through AMPK inhibition. Our data suggests that VTE may improve diet-induced NASH, which involves the pharmacological modulation of the AMPK-LXRα signaling pathway.

## Introduction

Non-alcoholic fatty liver disease (NAFLD) is a metabolic liver disorder of global prevalence (Friedman et al., 2018; Samuel and Shulman, 2018). It is estimated to affect approximately one in four adults worldwide (Younossi et al., 2018). Non-alcoholic steatohepatitis (NASH) is a more serious stage of NAFLD with associated steatosis, hepatocyte injury, inflammation, and different degrees of fibrosis; it may progress to cirrhosis and hepatocellular carcinoma (Chalasani et al., 2018; Sheka et al., 2020). The pathogenesis of NASH is complex, with many pathogenic factors: insulin resistance (IR), lipotoxicity, inflammation, and endoplasmic reticulum (ER) stress have all been implicated in the development of NASH (Friedman et al., 2018). However, details of NASH pathogenesis remain obscure. To date, no NASH-specific drug treatment has been approved.

The serine/threonine-protein kinase adenosine monophosphate-(AMP)-activated protein kinase (AMPK) is a crucial energy regulator which orchestrates several metabolic pathways and may have therapeutic importance in treating obesity, type 2 diabetes, and NAFLD (Garcia and Shaw, 2017; Steinberg and Carling, 2019). Diet-induced non-alcoholic fatty liver (NAFL) in mice exhibits reduced AMPK activity (Boudaba et al., 2018). Liver-specific AMPK deletion exaggerates liver injury in mice with NASH (Zhao et al., 2020). Numerous AMPK agonists have anti-NAFLD activity, suggesting AMPK activators may be potential treatments for NASH (Smith et al., 2016).

The nuclear hormone receptor liver X receptor α (LXRα) is a ligand-activated transcription factor with key roles in lipid and glucose homeostasis (Calkin and Tontonoz, 2012). Previous work has shown LXRα helps regulate energy metabolism in the fatty liver (Calkin and Tontonoz, 2012). LXRα activation promotes steatosis and fatty acid synthesis in the liver by elevating lipogenesis, leading to so-called “fatty liver” (Schultz et al., 2000). Animal studies and clinical trials show that LXRα-deficiency or inhibition of LXRα transcriptional activity reduces hepatic steatosis (Kalaany et al., 2005; Zhang et al., 2019). Prior work indicates that treatment using an LXR inhibitor can modulate hepatic fibrosis and inflammation in NASH (Griffett et al., 2015). Several natural products acting through LXR antagonism also have anti-NAFLD effect without obvious side-effect in rodents (Ni et al., 2019).

AMPK activation regulates LXRα transcriptional activity by inactivating LXRα and blocking endogenous LXRs ligand production in hepatocytes (Hwahng et al., 2009; Yang et al., 2009; Yap et al., 2011). An AMPK activator improves liver steatosis and inflammation in high fat (HF) diet-induced mice by depressing LXRα activity, which is typically mediated by AMPK (Hwahng et al., 2009). Targeting AMPK-LXRα signaling shows promise for NASH treatment.

Vine tea, a traditional Chinese medicinal herb and functional food, is widely consumed in southwest China (Xie et al., 2019). Several studies indicate that vine tea has diverse beneficial biological activities. These include antioxidant (Xie et al., 2019), anti-inflammation (Hou et al., 2015), and anti-sepsis effects (Liang et al., 2020), as well as hepatoprotection (Xie et al., 2020), and roles in glucose and lipid regulation (Xiang et al., 2019). Vine tea can be used to treat cough, fever, and metabolic diseases (Xiang et al., 2019; Xie et al., 2019). Although recent studies indicate vine tea, and its component dihydromyricetin (DMY), has beneficial effects on obesity, hyperglycaemia, hyperlipaemia, liver steatosis, and chemical-induced liver fibrosis *via* multiple biological targets (Tong et al., 2020), its role in controlling NASH and its pharmacological mechanism of action require further elucidation.

Here, we hypothesized that AMPK activation-mediated inhibition of LXRα activity is the principal pharmacological mechanism of vine tea extract (VTE). We investigated whether VTE protects against NASH and clarified whether the positive effects of VTE are associated with AMPK-LXRα signaling activation.

## Materials and Methods

### Chemicals and VTE Preparation

Dry Vine tea (Anhui Huangshan Greenxtract Co., Ltd.) was weighed 500 g and extracted using purified water (5 L) at 85°C for 2 h. The water extract collection was concentrated at 40°C with a rotary evaporator under reduced pressure and freeze-dried to a VTE powder. VTE powder was dissolved in dimethylsulfoxide (DMSO) to the final concentration of 500 mg ml^−1^ for cell culture. T0901317, Compound C, dihydromyricetin, myricetrin, and myricetin were purchased from Sigma–Aldrich (St. Louis, MO, United States) and dissolved in DMSO or water. High-fat diets (HFD, 60% of calories derived from fat), Chow diets (Chow, 10% of calories derived from fat) were purchased from Research Diet (D12492 and D12450B, New Brunswick, NJ, United States), Methionine and choline-deficient l-amino acid diets (MCDD, 20% of calories derived from fat) and methionine and choline supplement diet (MCSD) were purchased from Research Diet (A02082002B and A02082003B, New Brunswick, NJ, United States).

### Components of Analysis

The VTE was dissolved in water at 10 mg ml^−1^, while the standards of dihydromyricetin, myricetrin, and myricetin were dissolved in water and diluted to 1 μM. The chemicals profile of VTE and standards were analysed in a High-Performance Liquid Chromatography DAD system (Agilent 1290 HPLC). HPLC separation was conducted with an Agilent Zorbax EC plus (2.1 × 50 mm) maintained at 30°C at a flow rate of 0.3 ml min^−1^ with an injection volume of 1 μL and detection wavelength was 254 nm. Purified water containing 0.1% formic acid (solvent A) and acetonitrile containing 0.1% formic acid (solvent B) with a gradient system, which was 0–10 min, 95–80% A; 10–15 min, 10–95% A.

### Cell Culture and MTT Assay

HepG2 cells (obtained from ATCC) were cultured in high-glucose DMEM containing 10% FBS at 37°C in 5% CO_2_ and seeded on a 96-well plate (5,000 cells well^−1^) and incubated with control (0.1% DMSO) and VTE (0, 60, 125, 250, and 500 μg ml^−1^) for 24 h. Then, cells were stained with MTT (10 µL of 5 mg ml^−1^, Sigma–Aldrich) for 3 h, before DMSO (200 µL) was added and absorbance was measured on a Synergy H4 microplate reader (Biotek, Vermont, United States) at 570 nm and background absorbance measured at 630 nm. Background absorbance was subtracted from signal absorbance to obtain normalized absorbance values. The colourimetric signal obtained was proportional to the cell number.

### Transient Transfection and Luciferase Reporter Assays

The Dual-Luciferase Reporter Assay System (Promega, United States) was used to carry out the reporter assay as previously described (Huang et al., 2006).

For LXRα/β transcription activity assay, HepG2 cells were seeded on a 48-wells plate (1 × 10^6^ cells. well^−1^). After 80% growth confluence. Cells were co-transfected with the expression plasmids for the full-length human LXRα, *ß* (pCMX-hLXRα, β) and LXRE reporter vector (LXRE), ligand-binding domain (LBD) of human LXRα (pCMXGal-hLXRα-LBD) and the Gal4 reporter vector MH100 × 4-TK-Luc, and pREP7 (Renilla luciferase). After 24 h, cells were incubated with control (0.1% DMSO), VTE (125, 250, and 500 μg ml^−1^), T0901317 (5 μM), Compound C (5 μM), and DMY (10 and 20 μM) for another 24 h to determine luciferase activities.

To test NF-κB transcriptional activity, HepG2 cells were co-transfected with the p65 expression vector, NF-κB reporter vector (NF-κBx3-LUC), and pREP7. After 24 h, cells were pre-treated with the control (0.1% DMSO), VTE (250 and 500 μg ml^−1^) for 18 h before treatment with TNFα (10 ng ml^−1^) for 6 h. Subsequently, cells were collected and subjected to luciferase activity analysis.

All transfection experiments were conducted with FuGENE-HD (Roche) and a control plasmid pREP7 (Renilla luciferase) reporter was co-transfected for normalizing transfection efficiencies. The ratio of plasmid amount of 1 μg of the relevant expression plasmid combined with 1 μg of reporter plasmids and 0.1 μg of pREP7 was used for all transfection experiments. The transfection mixture contained 10 μg of total plasmids and 15 μL FuGENE-HD per ml of DMEM. All plasmids for gene reporter assay are provided by Dr. Saez.

### Animal and Drug Administration

All animal protocols used in this study were approved and conducted in accordance with the guidelines of the Animal Ethical Committee of Shanghai University of Traditional Chinese Medicine (Approval Number: SZY20150523). Male C57BL/6J mice (20 ± 2 g) were purchased from the SLAC Laboratory (Shanghai, China) and housed under the specific pathogen-free condition that controlled temperature (22–23°C) and a 12 h light, 12 h dark cycle.

For high-fat diet (HFD)-induced obese (DIO) mouse treatment, Male C57BL/6J mice were fed with HFD and water *ad libitum* for 16 weeks to induce metabolic disorders and fatty liver and these mice were then randomly divided into three groups according to body weight: chow group (10% of calories derived from fat), High-fat group (HF, 60% of calories derived from fat) and VTE treatment group (HF + VTE, HF diet supplemented with VTE powder, at a dose of 1 g 100 g^−1^ diet). Mice were treated for additional 6 weeks. The food intake amount was measured by recording food weight every 2 days throughout the experiment. The amount of food intake over a 24 h period was calculated.

For MCDD-induced NASH mouse treatment, Male C57BL/6J mice were managed as the same methods in HFD-fed experiment. Male mice (20 ± 2 g) were randomly divided into three groups according to body weight: MCS group (fed MCSD), MCD (fed MCDD) and VTE treatment group (MCD + VTE, MCDD supplemented with VTE powder, at a dose of 1 g 100 g^−1^ diet). Mice were fed with corresponding diets and water *ad libitum* for additional 6 weeks before the end of the experiment.

At the end of all animal experiments, mice were killed via anaesthetizing with 20% urethane (Sigma, St. Louis, MO) and drawing cardiac blood, and the livers were harvested for subsequent use in various assays as indicated.

### Serum Chemistry Analysis

At the final stage of animal experiment research, mice were anaesthetized with 20% urethane and cardiac blood was collected. Serum was obtained after centrifugation at 800 *g* for 10 min and separated subsequently 100 μL to determine the levels of serum alanine aminotransferase (ALT), aspartate transaminase (AST), triglyceride (TG), total cholesterol (TC), HDL cholesterol (HDL-c), and LDL cholesterol (LDL-c) by a Hitachi 7020 Automatic Analyser (Hitachi, Ltd., Tokyo, Japan).

### Hepatic Lipid Content Determination

The quantities of TC and TG in the liver was determined as described previously (Folch et al., 1957). In short, the liver tissue was homogenized and then extracted with equal volumes of chloroform-methanol. The chloroform phase was transferred to a new tube and dried. Isopropyl alcohol was used to resuspend the extraction. Lipid content (Kingha WK, China) in the liver was then assayed using kits by following the manufacturer’s protocols.

### Histochemistry

Liver tissues were fixed with 4% paraformaldehyde, and paraffin-embedded or frozen sections were cut at 5 μm thickness. Sections were stained with haematoxylin and eosin (HE) or Oil Red O following by a standard procedure.

### RNA Extraction and Quantitative Reverse Transcriptase PCR Analysis

Total RNA from mouse livers and HepG2 cells was isolated using the TRIzol method (Invitrogen, Carlsbad, CA). The first-strand cDNA was synthesized with a cDNA synthesis kit (Fermentas, Madison, WI). Quantitative real-time polymerase chain reaction (PCR) was carried out using SYBR Green PCR Mastermix. The results were analysed on an ABI StepOnePlus real-time PCR system (Applied Biosystems, United States) using the 2−^ΔΔCt^ method. Values were normalized to β-ACTIN. Sequences of primers were listed in Tables 1, 2.

**TABLE 1:** Sequences of the mouse primers used in real time PCR.

Gene	Sense primer	Anti-sense primer
β-ACTIN	TGT​CCA​CCT​TCC​AGC​AGA​TGT	AGC​TCA​GTA​ACA​GTC​CGC​CTA​GA
PPARγ	CGC​TGA​TGC​ACT​GCC​TAT​GA	AGA​GGT​CCA​CAG​AGC​TGA​TTC​C
FXR	TTC​CTC​AAG​TTC​AGC​CAC​AG	TCG​CCT​GAG​TTC​ATA​GAT​GC
SCD1	CTT​ATC​ATT​GCC​AAC​ACC​A	CTTCTCGGCTTTCAGGTC
TNFα	ATG​GAT​CTC​AAA​GAC​AAC​CAA​CTA​G	ACG​GCA​GAG​AGG​AGG​TTG​ACT​T
MCP-1	AGGTCCCTGTCATGCTTC	GTGCTTGAGGTGGTTGTG
IL1β	TCG​TGC​TGT​CGG​ACC​CAT​AT	GGT​TCT​CCT​TGT​ACA​AAG​CTC​ATG
IL6	AAC​CAC​GGG​CTT​CCC​TAC​TT	TCT​GTT​GGG​AGT​GGT​ATC​CTC​TGT
CYP7A1	TGA​TCC​TCT​GGG​CAT​CTC​AAG​CAA	AGC​TCT​TGG​CCA​GCA​CTC​TGT​AAT
CPT1a	TAT​GTG​AGT​GAC​TGG​TGG​GAG​GA	TAT​GGG​TTG​GGG​TGA​TGT​AGA​GC
CPT1β	TGG​GAC​TGG​TCG​ATT​GCA​TC	CAG​GGT​TTG​TCG​GAA​GAA​GAA​AA
CHOP	CTC​GCT​CTC​CAG​ATT​CCA​GTC	CTT​CAT​GCG​TTG​CTT​CCC​A
SREBP-1c	GGC​TAT​TCC​GTG​AAC​ATC​TCC​TA	ATC​CAA​GGG​CAT​CTG​AGA​ACT​C
FAS	CTG​AGA​TCC​CAG​CAC​TTC​TTG​A	GCC​TCC​GAA​GCC​AAA​TGA​G
ABCA1	GGC​AAT​GAG​TGT​GCC​AGA​GTT​A	TAG​TCA​CAT​GTG​GCA​CCG​TTT​T
ABCG1	TCC​CCA​CCT​GTA​AGT​AAT​TGC​A	TCG​GAC​CCT​TAT​CAT​TCT​CTA​CAG​A
F4/80	TGA​CTC​ACC​TTG​TGG​TCC​TAA	CTT​CCC​AGA​ATC​CAG​TCT​TTC​C
LXR a	GAG​TGT​CGA​CTT​CGC​AAA​TGC	CCT​CTT​CTT​GCC​GCT​TCA​GT
LXRβ	CAG​GCT​TGC​AGG​TGG​AAT​TC	ATG​GCG​ATA​AGC​AAG​GCA​TAC​T

**TABLE 2:** Sequences of the human primers used in real time PCR.

Gene	Sense primer	Anti-sense primer
β-ACTIN	AAT​CTG​GCA​CCA​CAC​CTT​CTA	ATA​GCA​CAG​CCT​GGA​TAG​CAA​C
SCD1	GCC​CCT​CTA​CTT​GGA​AGA​CGA	AAG​TGA​TCC​CAT​ACA​GGG​CTC
CYP7A1	GAG​AAG​GCA​AAC​GGG​TGA​AC	AGC​ACA​GCC​CAG​GTA​TGG​A
SREBP-1c	GGA​TTG​CAC​TTT​CGA​AGA​CAT​G	AGG​ATG​CTC​AGT​GGC​ACT​G
FAS	AGA​CAC​TCG​TGG​GCT​ACA​GCA​T	ATG​GCC​TGG​TAG​GCG​TTC​T
ABCA1	GGA​AGA​ACA​GTC​ATT​GGG​ACA​C	GCT​ACA​AAC​CCT​TTT​AGC​CAG​T
ABCG1	ATT​CAG​GGA​CCT​TTC​CTA​TTC​GG	CTC​ACC​ACT​ATT​GAA​CTT​CCC​G
LXR a	CCT​TCA​GAA​CCC​ACA​GAG​ATC​C	ACG​CTG​CAT​AGC​TCG​TTC​C

### Western Blotting

For intracellular AMPK activity analysis, HepG2 cells were seeded in six-well plates and grown to 50% confluence with high-glucose DMEM followed by a 6 h free-serum quench. Then, cells were treated with VTE (0, 60, 125, 250, and 500 μg ml^−1^) for 24 h. Then cells were collected for Western blotting.

The whole-protein extractions from HepG2 cell and livers of mice were separated using 10% SDS-PAGE and then transferred to nitrocellulose membranes. The expression of protein was subsequently probed with primary antibodies of AMPK (Affinity Biosciences, AF6423), phosphorylation of AMPK (p-AMPK, Affinity Biosciences, AF3423), Cleaved Caspase-3 (cle-CASP3, Affinity Biosciences, AF7022), CHOP (Proteintech Group, 15204-1-AP) and β-ACTIN (Proteintech Group, 60008-1-Ig) with 1:1,000 dilution in 5% bovine albumin at 4°C overnight followed by HRP-labelled secondary antibodies (1:10,000, Yeasen, Shanghai, China) for 1 h at room temperature. The protein bands were detected and analysed using the Tanon-5200 Multi-image detection system and ImageJ software respectively. The levels of relative protein were normalized to β-ACTIN.

### Enzyme-Linked Immunosorbent Assay

Whole trunk blood was obtained and then centrifuged for 15 min at 1,500 g and 4°C to separate serum. Levels of serum TNFα (Westang Bio-Tech, Shanghai, China) in mice were measured using an ELISA kit following by the corresponding kit protocol with a Synergy H4 microplate reader.

### Immunofluorescence Staining

The sections of liver from MCSD- and MCDD-fed mice were probed with primary antibodies of F4/80 (1:100, Affinity Biosciences, DF2789) at 4°C overnight followed by Alexa Fluor 594-labelled secondary antibodies (1:600, Yeasen, Shanghai, China) for 1 h at room temperature. Nuclei were counterstained with DAPI (0.5 μg ml^−1^, Yeasen, Shanghai, China).

### Terminal Deoxynucleotidyl Transferase dUTP Nick End Labeling Assay

The sections of the liver from MCSD- and MCDD-fed mice were subjected to TUNEL analysis by using an Alexa Fluor 640 TUNEL apoptosis detection kit (Yeasen, Shanghai, China) according to the manufacturer’s protocol. Nuclei were counterstained with DAPI (0.5 μg ml^−1^, Yeasen, Shanghai, China).

### Statistical Analysis

All values are expressed as means ± SEM and were analysed using the statistical software package for social science (SPSS, version 15.0). Differences between the mean values of the treatment group and control or among more than two groups were detected using Student’s paired or unpaired two-tailed t-tests or ANOVA respectively. Differences with *p* values < 0.05 were considered to be statistically significant.

## Results

### VTE Protects Against Fatty Liver in DIO Mice

**FIGURE 1:**
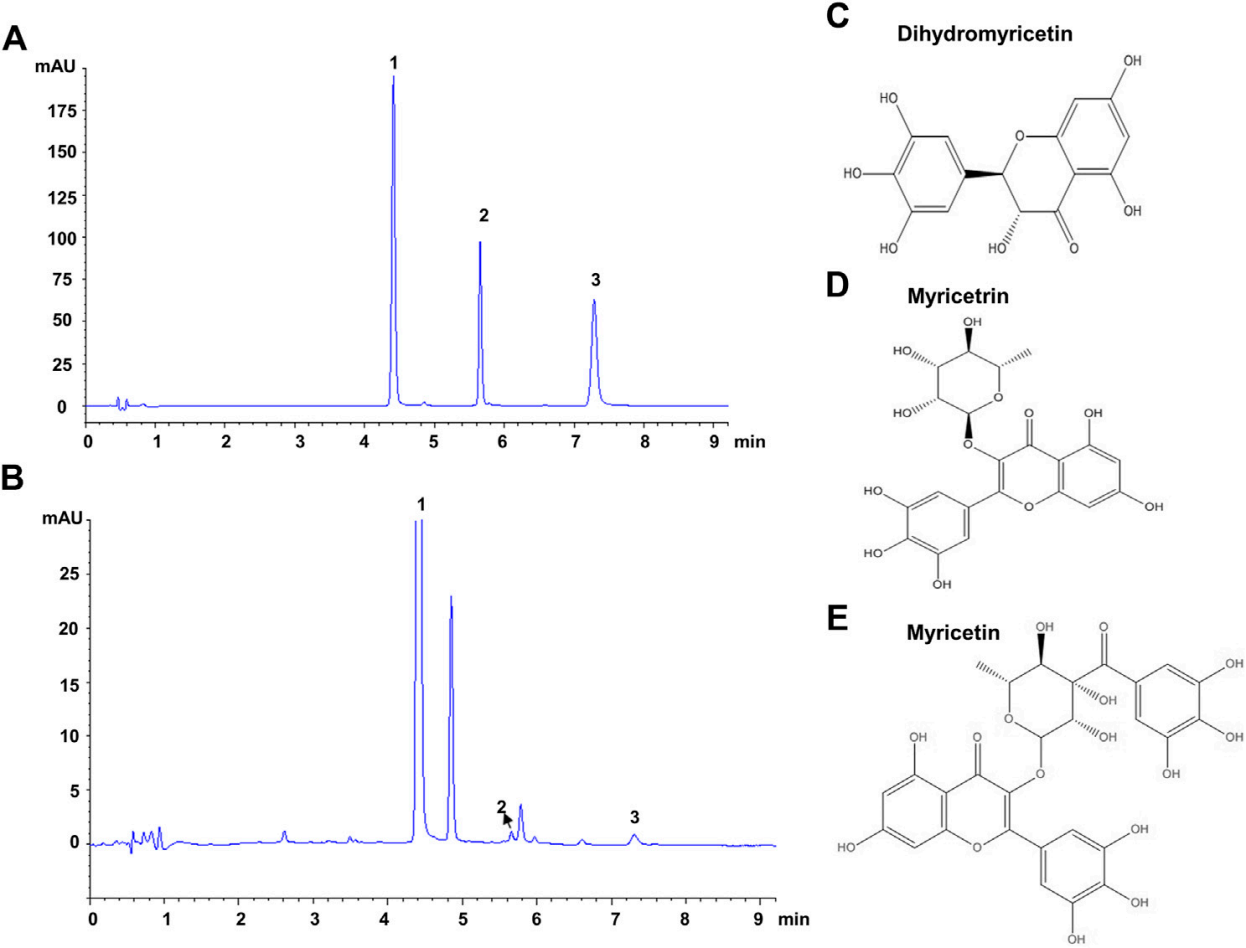
HPLC chromatograms of VTE **(A)** HPLC chromatogram of standards species of dihydromyricetin, myricetrin, and myricetin **(B)** HPLC chromatogram of VTE detected at 254 nm **(C)** Dihydromyricetin **(D)** Myricetrin **(E)** Myricetin.

The composition of VTE was evaluated using HPLC. This indicated DMY was present in large quantities and accounted for approximately 53.5% of the total content of VTE (Figures 1A,B and Table 3). Myricetrin and myricetin were also present in VTE (Figures 1A,B and Table 3).

**TABLE 3:** Linear regression and amounts determination of main compounds in VTE.

Analytes	Calibration curves	R^2^	Content (mg.g^−1^)	Test range (μg.ml^−1^)
Dihydromyricetin	y = 6.5180 × ^−^2.4757	0.9999	535.50	20–200
Myricetrin	y = 2.6793 × ^−^2.2672	0.9999	7.92	2–20
Myricetin	y = 3.7293 × ^−^9.3609	0.9999	14.27	2–20

**FIGURE 2:**
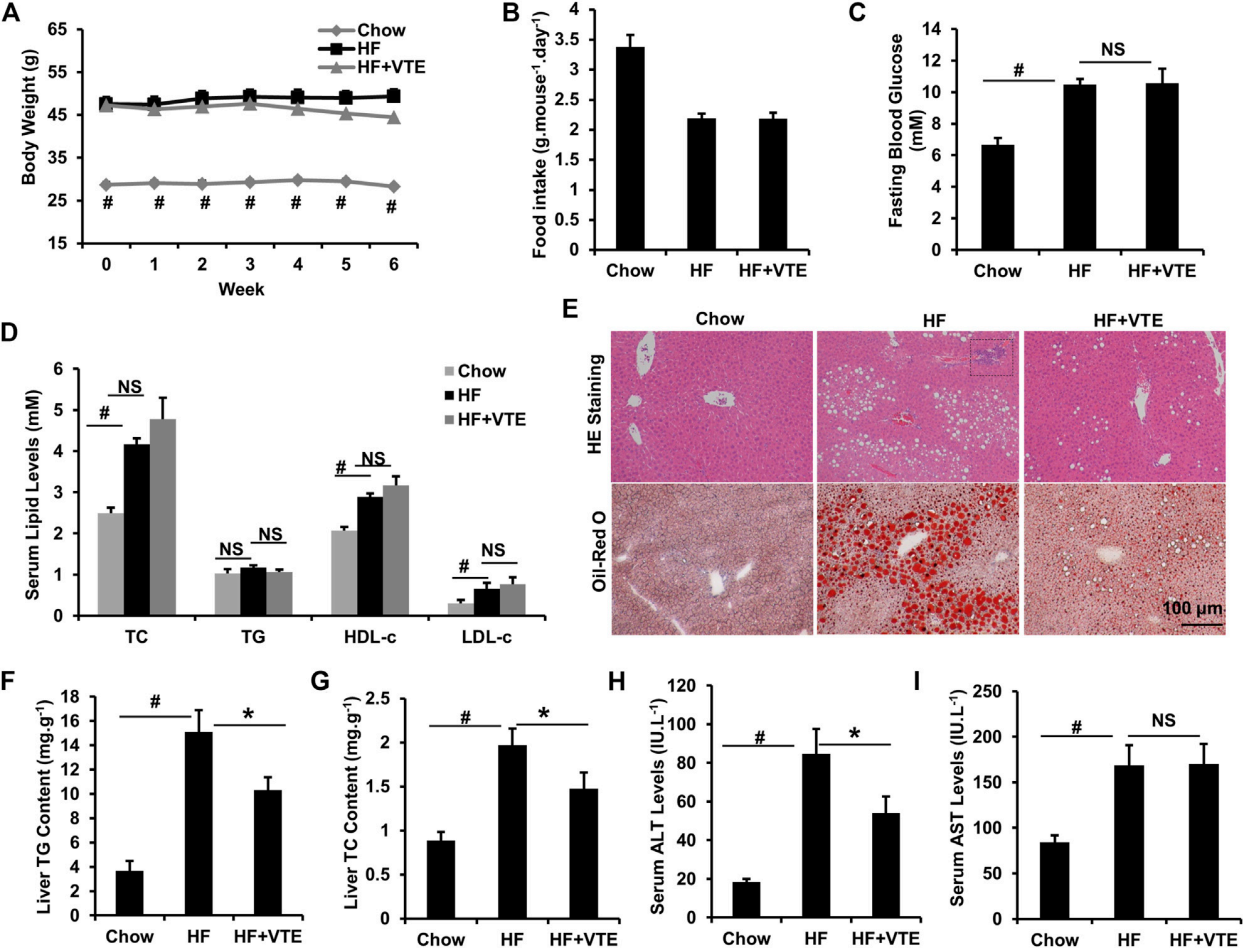
VTE protects DIO mice from NAFLD. Obese C57BL/6J mice were fed with an HFD (high-fat diet) supplemented with or without VTE (1 g 100 g^−1^ diet) for 6 weeks **(A)** Body weight was recorded during the 6-weeks treatment **(B)** Food intake amount **(C)** Fasting blood glucose **(D)** Serum total cholesterol (TC), triglyceride (TG), high-density lipoprotein cholesterol (HDL-c), low-density lipoprotein cholesterol (LDL-c) levels **(E)** Haematoxylin and eosin (H and E) and Oil Red O staining of livers from the chow diet-, HF diet-, and HF + VTE-fed mice. Scale bars, 100 μm **(F)** Liver TG levels **(G)** Liver TC levels **(H)** Serum ALT levels **(I)** Serum AST levels. Data are statistically analysed as means ± SEM (*n* = 6 per group). ^#^ *p* < 0.05, HF versus Chow group. *p < 0.05, HF versus HF + VTE group. NS, no significance. Dotted rectangles denote hepatic inflammatory cell infiltration.

Next, we investigated the protective action of VTE in DIO mice. VTE treatment (1 g VTE 100 g^−1^ diet) decreased body-weight gain in HFD-fed obese mice during the 6-weeks treatment, although no statistically significant difference was observed (Figure 2A). Food intake measurements indicated that VTE did not alter feeding behaviour in DIO mice (Figure 2B). Analysis of serum metabolic profiles indicated VTE did not correct the elevated fasting blood sugar levels and dyslipidemia seen in DIO mice, as shown by measured levels of TC, TG, HDL-c, and LDL-c in Figures 2C,D.

To determine VTE’s protective effects on the fatty liver, liver sections from chow control, HF control, and VTE-treated mice were examined using HE analysis and Oil-red O staining. VTE treatment reduced HFD-induced hepatic steatosis and inflammatory cell infiltration (Figure 2E). In support of histopathology observations, hepatic lipid content determinations indicated HFD feeding markedly increased TG and TC levels in the liver of DIO mice when compared to chow controls, which was not seen in VTE-treated obese mice (Figures 2F,G). To determine if VTE treatment improves injured liver function in DIO mice, we tested serum ALT and AST activities in chow controls, HF controls, and VTE treated mice. Figures 2H,I indicate higher levels of serum ALT and AST in HFD-fed control mice when compared to chow-fed mice, reflecting increased hepatic injury in obese mice. 6 weeks of VTE treatment reversed increased ALT, but not AST, levels. This indicates VTE may protect against liver damage in the fatty liver in HFD mice.

### VTE Ameliorates NASH in MCDD-Fed Mice

**FIGURE 3:**
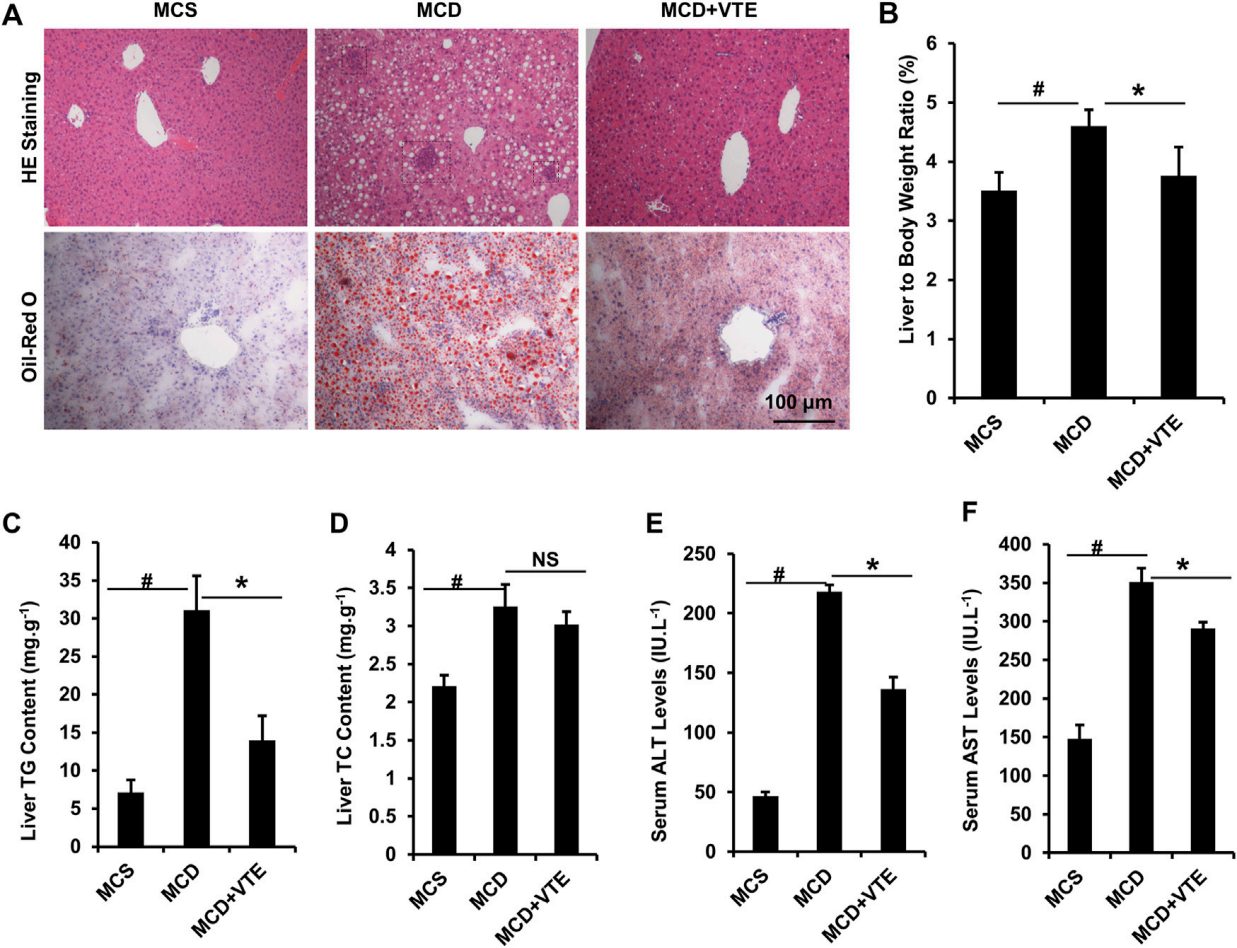
VTE resists to NASH in MCDD-fed mice. Male C57BL/6J mice (20 ± 2 g) were fed an MCD diet in the presence and absence of VTE (1 g 100 g^−1^ diet) for 6 weeks **(A)** Haematoxylin and eosin (H and E) and Oil Red O staining of livers from the MCS diet-, MCD diet- and MCD + VTE-fed mice. Scale bars, 100 μm **(B)** The ratio of liver to bodyweight of the mice **(C, D)** The quantitative results of TG and TC content in livers **(E)** Serum ALT levels **(F)** Serum AST levels. The data are presented as mean ± SEM. *n* = 5−6 for all groups. ^#^ *p* < 0.05, MCD compared with the MCS group. *p < 0.05, MCD compared with the MCD + VTE group. NS, no significance. Dotted rectangles denote hepatic inflammatory cell infiltration.

NASH damage to mouse livers resulting from HFD feeding is relatively mild. To corroborate the beneficial effects of VTE, MCDD-model NASH mice were subjected to VTE treatment. Hepatic histopathological analyses and liver/body weight index indicated that extensive steatosis, inflammatory cell infiltration (Figure 3A), and increased liver/body weight ratios (Figure 3B) in livers from MCDD-fed mice, when compared to MCS controls, were almost completely prevented by 6-weeks VTE treatment (Figures 3A,B). This was supported by decreased TG levels, but not TC levels, in VTE-treated mice, when compared to MCDD-fed controls, according to hepatic lipid content (Figures 3C,D). MCDD feeding notably increased serum ALT and AST levels when compared to MCSD-fed controls (Figures 3E,F). This suggests liver function was severely impaired, which was effectively protected by VTE treatment, as indicated by reductions in serum ALT and AST levels in VTE-treated controls when compared to MCD controls (Figures 3E,F). This indicates that VTE may protect mice from MCDD-induced hepatic steatosis, inflammation, and resulting damage.

**FIGURE 4:**
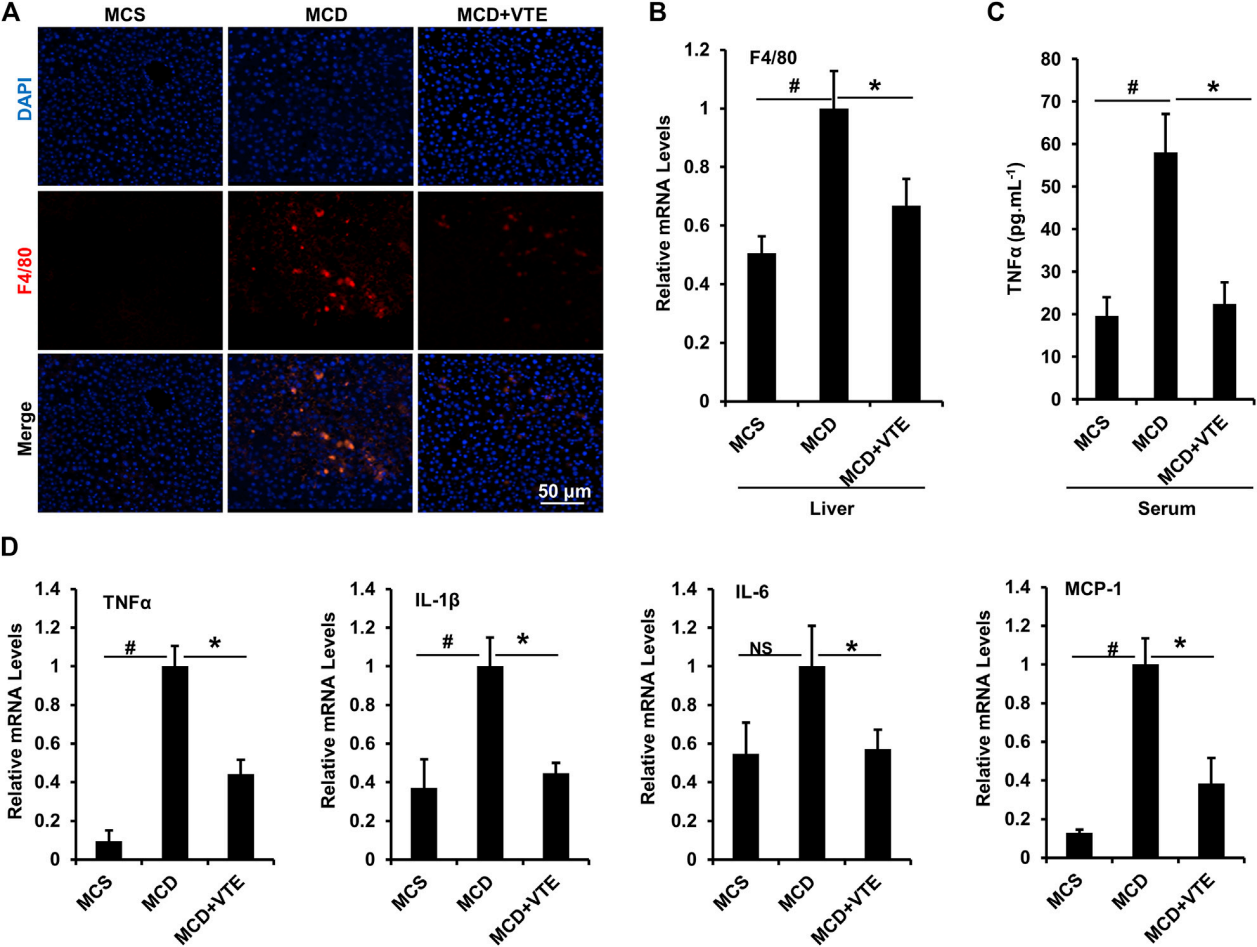
VTE inhibits hepatic inflammation in MCDD-fed mice. Male C57BL/6J mice (20 ± 2 g) were fed an MCD diet in the presence and absence of VTE (1 g 100 g^−1^ diet) for 6 weeks **(A)** F4/80 immunofluorescence analysis in the livers from the MCS diet-, MCD diet- and MCD + VTE-fed mice. Scale bars, 50 μm **(B)** F4/80 mRNA levels in livers; *n* = 5 per group **(C)** ELISA analysis of serum TNFα levels; *n* = 5–6 per group **(D)** Relative mRNA expression of pro-inflammation genes in mouse liver; *n* = 3–5 per group. β-ACTIN was used as an internal control for normalizing the mRNA levels. Data are statistically analysed as means ± SEM. ^#^ *p* < 0.05, MCD compared with the MCS group of mice. *p < 0.05, MCD compared with the MCD + VTE group. NS, no significance.

Expression of macrophage marker F4/80 correlates positively with macrophage infiltration (Waddell et al., 2018). F4/80 immunostaining in livers from MCDD-model mice indicated that MCDD feeding increased hepatic expression of F4/80 compared to MCSD-fed controls (Figure 4A). This indicates exaggerated inflammatory cell infiltration. VTE reversed such induction (Figure 4A). Subsequent determination of liver F4/80 mRNA levels supports this finding (Figure 4B). We also analysed mouse serum pro-inflammatory cytokine TNFα levels. As assessed using ELISA, VTE suppressed upregulated TNFα levels in MCDD-fed mice (Figure 4C). To verify the effect of VTE on inhibiting liver inflammation, hepatic pro-inflammatory cytokine expression was assayed. MCDD feeding significantly increased the relative mRNA levels of hepatic TNFα, IL-1β, IL-6, and MCP-1, when compared to MCS controls (Figure 4D). VTE treatment markedly decreased mRNA levels corresponding to the four pro-inflammatory genes in MCDD-fed mice (Figure 4D), supporting our hypothesis that VTE is able to decrease diet-induced steatohepatitis in mice.

### VTE Attenuates Apoptosis and Endoplasmic Reticulum Stress in the Liver of MCDD-Induced NASH Mice

**FIGURE 5:**
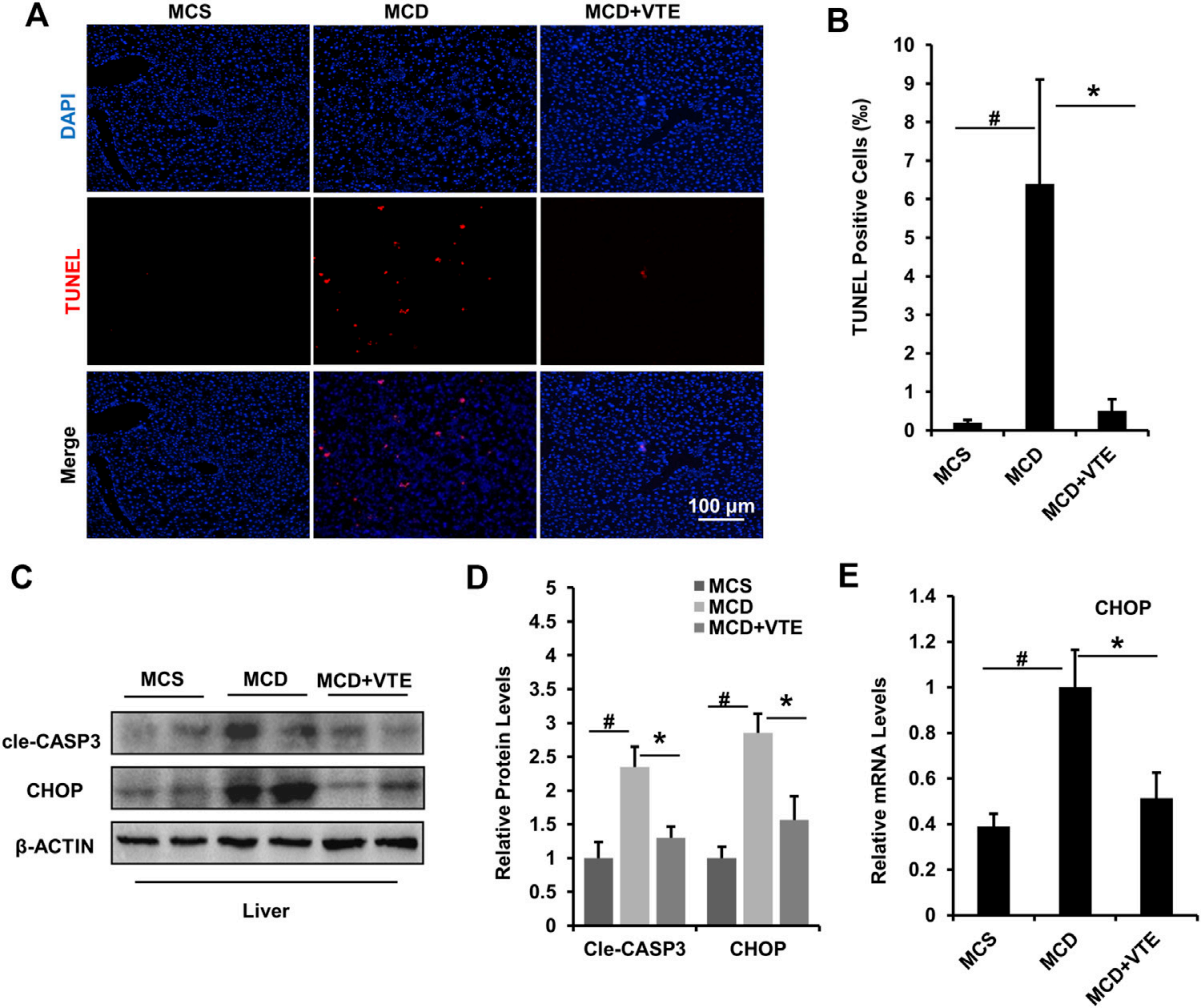
VTE relieves apoptosis and ER stress in the livers of MCD-induced mice. Male C57BL/6J mice (20 ± 2 g) were fed an MCD diet in the presence and absence of VTE (1 g 100 g^−1^ diet) for 6 weeks **(A)** TUNEL staining in the livers from the MCS diet-, MCD diet-, and MCD + VTE-fed mice. Scale bars, 100 μm **(B)** TUNEL positive cells (‰). Data are statistically analysed as means ± SEM (*n* = 6 per group) **(C, D)** Protein levels of cle-CASP3 and CHOP in mice were analysed by immunoblotting. The relative protein level was shown **(E)** The relative mRNA expression of CHOP in mouse liver. β-ACTIN was used as an internal control for normalizing the mRNA levels and protein levels. *n* = 5 for all groups. ^#^ *p* < 0.05, MCD compared with the MCS group. *p < 0.05. MCD compared with the MCD + VTE group.

Hepatic apoptosis and ER stress induced in NASH causes progressive injury, fibrosis, and cirrhosis in the liver (Friedman et al., 2018). Here, we analysed hepatic apoptosis and ER stress in MCDD-fed mice which exhibited a more severe NASH liver damage than that in HFD-fed mice. As detected using TUNEL staining, an increased TUNEL signal indicated MCDD feeding raised the number of apoptotic cells in mouse livers, when compared to MCSD-fed controls. NASH liver damage was almost completely suppressed by VTE treatment (Figures 5A,B).

Cle-CASP3 representing active caspase-3 has a key role in promoting apoptosis (McIlwain et al., 2013). CHOP-mediated ER stress also has important pro-apoptosis effects (Dai et al., 2016). VTE decreased elevated protein levels of cle-CASP3 and CHOP in MCDD-fed mice when compared to MCS controls (Figures 5C,D), which agrees with the difference in hepatic CHOP mRNA expression (Figure 5E). Thus suggests that VTE has a beneficial effect on anti-apoptosis and anti-ER stress in NASH, further supporting the protective effects of VTE in NASH treatment.

### VTE Activates AMPK-LXRα Signaling in Liver

**FIGURE 6:**
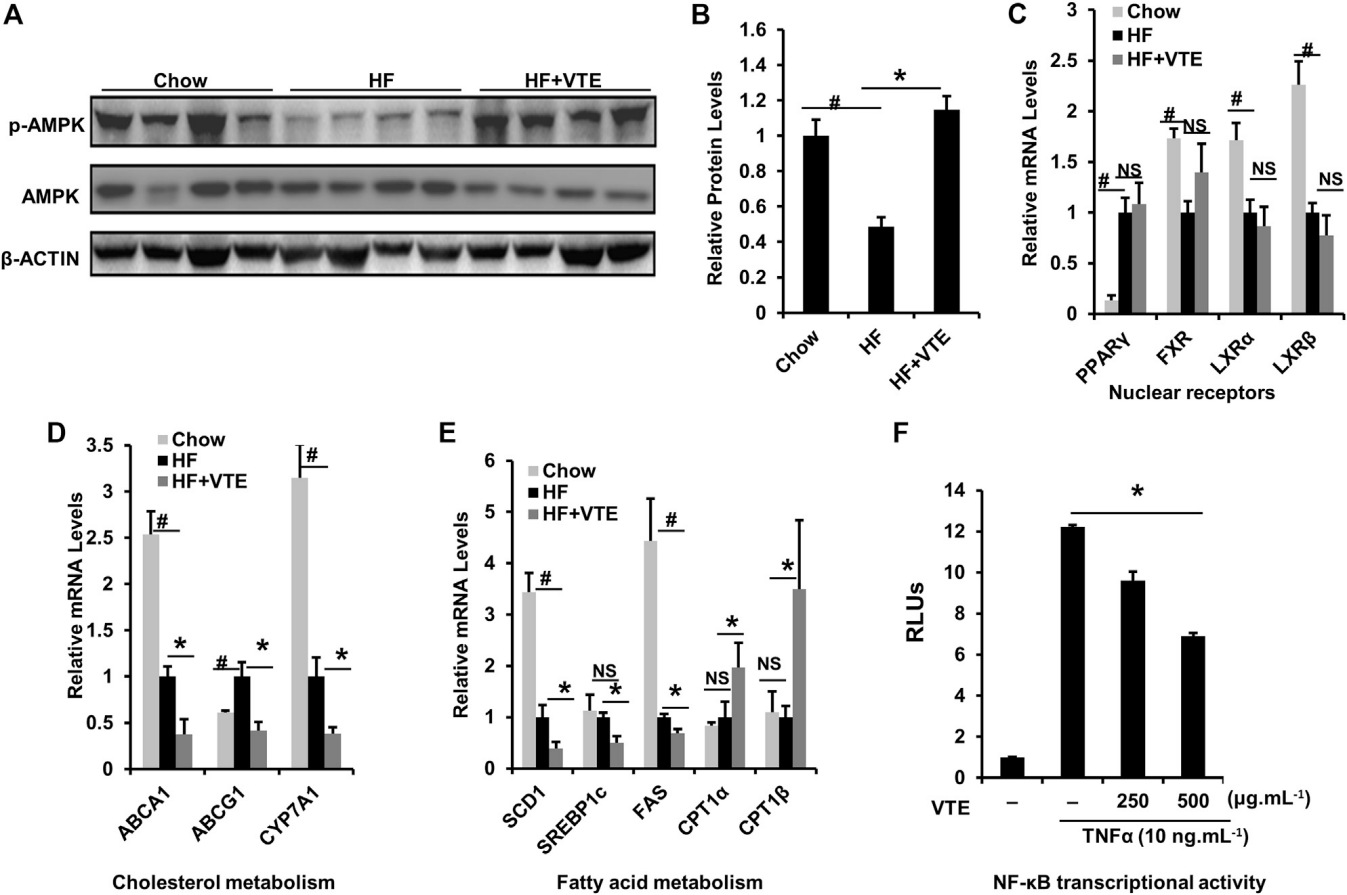
VTE ameliorating NASH involves AMPK-LXRα signaling activation. Livers from chow group mice and DIO mice fed with an HFD supplemented with or without VTE (1 g 100 g^−1^ diet) for 6 weeks were harvested and subjected to mRNA and immunoblotting analysis **(A, B)** Protein levels of p-AMPK and AMPK in DIO mice were analysed by immunoblotting. The relative protein level was shown. AMPK was used as a control for protein levels; *n* = 5 per group **(C–E)** Relative mRNA expression of hepatic genes; *n* = 3–5 per group. β-ACTIN was used as an internal control for normalizing the mRNA levels and protein levels. ^#^ *p* < 0.05, HF versus Chow group. *p < 0.05, HF versus HF + VTE group. NS, no significance **(F)** Hepatocellular NF-κB activity was analysed using a reporter assay. HepG2 cells were co-transfected with the p65 expression vector and NF-κBx3-LUC for 24 h and treated with the control (0.1% DMSO), VTE (250 and 500 μg ml^−1^) for another 18 h. Then cells were treated with TNFα (10 ng ml^−1^) for an additional 6 h. The relative luciferase units (RLUs) were measured by comparison to renilla luciferase activities. The results represent three independent experiments, and data are statistically analysed as means ± SEM (*n* = 3). *p < 0.05, versus vehicle control.

AMPK-LXRα signaling has an important regulatory role in liver energy metabolism (Garcia and Shaw, 2017). To assess if VTE improves NASH by activating AMPK-LXRα signaling, levels of AMPK and p-AMPK protein in DIO treated livers were determined. Hepatic phosphorylation of AMPK decreased by HFD feeding was restored by VTE treatment (Figures 6A,B), indicating hepatic AMPK activity is enhanced by VTE. As compared with chow controls, HFD-feeding upregulated the hepatic mRNA expression of PPARγ and ABCG1, but reduced FXR, LXRα, LXRβ, ABCA1, CYP7A1, SCD1, and FAS, without changing mRNA levels of SREBP1c and CPT1α, *ß* in mouse livers (Figures 6C–E). VTE did not alter the mRNA expression of nuclear receptor genes PPARγ, FXR, LXRα, or LXRβ (Figure 6C), but did decrease LXRα target genes ABCA1, ABCG1 and CYP7A1, SCD1, SREBP1c, and FAS in DIO mice livers (Figures 6D,E). These genes variously control for cholesterol transport and *de novo* synthesis, and lipogenesis (Calkin and Tontonoz, 2012). AMPK activation can promote intracellular fatty acid oxidation (Garcia and Shaw, 2017). We also noted that VTE remarkably up-regulated expression of hepatic fatty acid oxidation genes CPT1α and CPT1β (Figure 6E).

NF-κB is an important transcription factor controlling inflammatory responses (Catrysse and van Loo, 2017). Its transcriptional activity has been reported to be inhibited by AMPK activation (Greer et al., 2007). When testing NF-κB transcriptional activity, we observed that VTE (250 and 500 μg ml^−1^) suppressed NF-κB activation induced by TNFα incubation in HepG2 cells (Figure 6F), further supporting the role of VTE as an AMPK activator. These results suggest that AMPK-LXRα signaling activation is among mechanisms underlying NASH treatment by VTE.

### VTE Inhibits LXRα Transcriptional Activity *via* AMPK Activation *in vitro*

**FIGURE 7:**
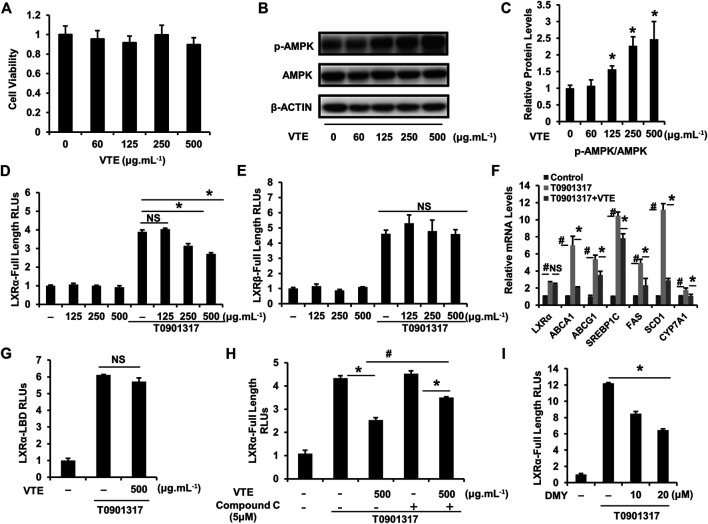
VTE stimulates intracellular AMPK-LXRα signaling **(A)** The MTT assay in HepG2 cell. HepG2 cells were treated with DMSO control and VTE (0, 60, 125, 250, and 500 μg ml^−1^) for 24 h and cytotoxicity was assessed by MTT. Data are statistically analysed as means ± SEM (*n* = 5) **(B, C)** Protein levels of p-AMPK and AMPK in HepG2 cells were analysed by immunoblotting. The relative protein level was shown. AMPK was used as a control for protein levels. **(D, E)** LXRα, *ß* transcriptional activities were analysed using transient transfection reporter assays. HepG2 cells were co-transfected with pCMX-hLXRα, *ß* and the LXRE reporter vector for 24 h and treated with control (0.1% DMSO), VTE (125, 250, and 500 μg ml^−1^), T0901317 (5 μM) for another 24 h. The relative luciferase units (RLUs) were measured by comparison with renilla luciferase activities **(F)** The relative mRNA levels of LXRα and its targets in VTE-treated and con (0.1% DMSO) HepG2 cells. HepG2 cells were incubated with VTE (500 μg ml^−1^) or/and T0901317 (2 μM) for 24 h β-ACTIN was used as an internal control for normalizing the mRNA levels **(G)** HepG2 cells were co-transfected with pCMXGal-hLXRα-LBD and MH100 × 4-TK-Luc for 24 h and treated with control (0.1% DMSO), VTE (500 μg ml^−1^), T0901317 (5 μM) for another 24 h. The relative luciferase units (RLUs) were measured by comparison with renilla luciferase activities **(H, I)** HepG2 cells were co-transfected with pCMX-hLXRα and the LXRE reporter vector for 24 h and treated with control (0.1% DMSO), VTE (500 μg ml^−1^), T0901317 (5 μM) Compound C (5 μM) and DMY (10 and 20 μM) for another 24 h. The relative luciferase units (RLUs) were measured by comparison to renilla luciferase activities. The results represent three independent experiments, and data are statistically analysed as means ± SEM (*n* = 3). *p < 0.05, control versus VTE group, T0901317 versus T0901317 + VTE group, T0901317 + Compound C + VTE versus T0901317 + Compound C group, or T0901317 versus T0901317 + DMY group. ^#^ *p* < 0.05, T0901317 compared with the control group, or T0901317 + VTE compared with the T0901317 + Compound C + VTE group. NS, no significance.

To further confirm the effect of VTE on activating AMPK-LXRα signaling, HepG2 cells were treated with VTE. No clear VTE-induced cytotoxicity was observed (Figure 7A). VTE at doses of 125, 250 and 500 μg ml^−1^ notably increased AMPK phosphorylation levels (Figures 7B,C), indicating VTE could enhance AMPK activity in hepatocytes.

Next, we investigated whether VTE could influence LXRα, *ß* transcription activity. Relative Luciferase Units (RLUs) from full-length LXRα, *ß* expression plasmids were obtained. Results indicate VTE (250 and 500 μg ml^−1^) remarkably inhibited LXRα transactivity induced by agonist T0901317 (Figure 7D), but not LXRβ transactivity (Figure 7E). mRNA expression also indicated that VTE (500 μg ml^−1^) significantly suppressed the main LXRα target gene ABCA1, ABCG1, SREBP1c, FAS, SCD1, and CYP7A1 in T0901317-induced HepG2 cells (Figure 7F), indicating the inhibition of LXRα signaling by VTE. To understand if VTE antagonized LXRα transcription activity via direct binding to LXRα, we tested luciferase transcriptional activity of LXRα-LBD. VTE exhibited no antagonism of T0901317-induced LXRα-LBD transactivity activation in HepG2 cells (Figure 7G). This suggests VTE does not inhibit LXRα activity by binding to the LBD of LXRα.

To determine if AMPK activation mediates VTE-induced LXRα antagonism, AMPK inhibitor Compound C was used to test LXRα transcription activity. Interestingly, Compound C co-incubation with VTE notably blunted VTE antagonism of LXRα activation in HepG2 cells (Figure 7H). Moreover, DMY also inhibited LXRα transactivity induced by T0901317 (Figure 7I). This indicates VTE may inhibit LXRα activity through AMPK activation.

## Discussion

The role of vine tea in controlling NASH and its underlying mechanism-of-action both remain unclear. This study indicates that VTE improves hepatic steatosis and liver injury in HFD-fed C57BL/6 mice, and decreases hepatic lipid accumulation, inflammatory cell infiltration, apoptosis, ER stress, and impaired liver function in MCDD-fed C57BL/6 NASH mice. Investigation of the pharmacological mechanism suggests that VTE enhances AMPK activity and inhibits LXRα signaling in NASH mouse livers. VTE depresses LXRα transactivity, which is associated with its AMPK activation, and that VTE suppresses NF-κB activation in hepatocytes. Our results confirm the effect of VTE when treating diet-induced NASH. AMPK-LXRα signaling activation may contribute to the beneficial effects of VTE on NASH.

Vine tea and isolated DMY are reported to exert anti-obesity, anti-dyslipidemia, and anti-hepatosteatosis effects in HFD-fed rodents (Xiang et al., 2019; Zeng et al., 2019). Here, our analysis of VTE components indicated that DMY was the principal component of VTE. In animal studies, we found VTE treatment inhibited body weight gain in HFD-fed mice, although this effect was not statistically significant. However, VTE did not alter fasting blood glucose and serum lipid levels in DIO mice. Consistent with previous work, VTE alleviated hepatic steatosis, decreased liver inflammatory cell infiltration, and protected the liver from injury. This was evidenced by improved hepatic pathologic phenotypes, reduced liver TG and TC levels, and lowered serum ALT levels in HFD-fed mice. This suggests VTE may have potential therapeutic effects in NASH treatment.

We explored the anti-NASH effect of VTE on MCDD-induced NASH mice. VTE almost completely blocked NASH progression, improving impaired liver function in MCDD-fed mice. Moreover, the well-established steatohepatitis phenotype in MCDD mice was confirmed by increased expression of macrophage marker F4/80. DMY has also been reported to be anti-inflammatory *in vivo* (Hou et al., 2015). Our data indicate VTE decreased F4/80 expression in mouse NASH livers, which supports the pathological observation of decreased inflammatory cell infiltration in livers from NASH mice after VTE treatment. We also found VTE treatment decreased pro-inflammatory cytokine TNFα protein levels in serum and TNFα, IL-1β, IL-6, and MCP-1 mRNA levels in livers from MCDD-fed mice. This supports the view that VTE can reduce steatohepatitis in NASH mice.

Cellular apoptosis and ER stress in liver are significant contributors to NASH pathogenesis (Friedman et al., 2018). Reducing cellular death and ER stress in liver shows promise as a treatment for NASH. Caspases are key aspartic-serine proteases promoting cell apoptosis (McIlwain et al., 2013). CHOP is a key driver of ER stress and cell apoptosis (Hetz et al., 2020). We found that VTE treatment prevented extensive apoptosis and increased gene expressions of cle-CASP3 and CHOP in mouse livers with MCDD-induced NASH, suggesting the VTE’s protective effects against hepatic death and ER stress in NASH.

AMPK activation and depression of LXRα activity are effective therapeutic approaches to protect against obesity, lipid metabolic disorder, and NASH (Griffett et al., 2015; Garcia and Shaw, 2017; Zhao et al., 2020). Liver-specific AMPK knockout exaggerates NASH liver damage, whereas AMPK activation could protect against liver damage and fibrosis due to NASH by inhibiting caspases and CHOP (Dai et al., 2016; Zhao et al., 2020). LXRα activity is reported to be regulated negatively by AMPK activation (Hwahng et al., 2009; Yap et al., 2011). AMPK activators have inhibitory effects on LXRα-mediated lipogenesis and hepatic steatosis (Hwahng et al., 2009). Thus, activating the AMPK-LXRα pathway may be a useful therapy in NASH. Additionally, DMY has been reported to alleviate hepatic steatosis *via* AMPK activation (Zeng et al., 2019). Interestingly, altered hepatic expression of cle-CASP3 and CHOP by AMPK activators aligns strongly with the reduced gene expression we observed in VTE-treated NASH mouse livers. Thus, we may hypothesize that vine tea is a modulator of AMPK-LXRα signaling and protects against NASH.

We successively analysed the hepatic protein levels of pAMPK and AMPK in DIO mice. The altered pAMPK/AMPK protein ratio in HFD-fed mouse livers were not observed in VTE-treated DIO mice, suggesting restoration of impaired AMPK activity after VTE treatment. Therefore, we examined mRNA expression of hepatic genes in AMPK-LXRα signaling in NASH mice. LXRα inhibition down-regulates the transcriptional levels of ABCA1, ABCG1, CYP7A1, SCD1, SREBP1c, and FAS directly. Such decreases in hepatic mRNA expression in HFD-fed (Figures 6D,E) and MCDD-fed mice (Supplementary Figure S2) were induced by VTE treatment. VTE also up-regulated CPT1α and CPT1β mRNA levels, which are also induced *via* AMPK activation. These genes are involved in cholesterol transport and conversion, lipogenesis, and fatty acid oxidation (Calkin and Tontonoz, 2012; Garcia and Shaw, 2017). Furthermore, AMPK is thought to be a negative regulator of pro-inflammatory transcription factor NF-κB (Greer et al., 2007). Previous work suggests vine tea can inhibit NF-κB transcriptional activity (Chen et al., 2018). This aligns strongly with our work, which demonstrates NF-κB inactivation. This is seen in VTE *in vitro* reporter assays, which indicate NF-κB inhibition is associated with NASH protection mediated by VTE. These results may help explain why VTE improves NASH, as well as suggesting AMPK-LXRα signaling activation may participate in the anti-NASH effects of VTE.

*In vitro* assays indicate that VTE increased the pAMPK/AMPK ratio in HepG2 cells, consistent with the activation of AMPK by VTE treatment in hepatocytes. Gene reporter and mRNA expression assays indicated that VTE treatment markedly inhibited LXRα transactivation and LXRα target genes ABCA1, ABCG1, SREBP-1c, FAS, SCD1, and CYP7A1, but not the transcriptional activity of LXRβ in condition of T0901317 stimulation. This indicates that VTE has a role in LXRα suppression. VTE was not observed acting as an antagonist in LXRα-LBD transactivity, indicating that it blocks LXRα transactivation indirectly. Furthermore, the inhibition of LXRα transactivation by VTE was blunted by compound C, implicating VTE-mediated reduction of LXRα activity involving AMPK activation. Moreover, DMY incubation also decreased transactivation of LXRα in HepG2 cells, supporting the role of LXRα antagonism in VTE activity. It is noted that compound C did not completely abrogated the action of LXRα inhibition by a high dose VTE, hinting other mechanisms may be involved in VTE’s antagonistic effects on LXRα. Additionally, we did not explore whether the anti-NASH effects of VTE is dependent on AMPK in this study. Further studies are warranted to specifically address these possibilities. Here, our data indicates VTE’s inhibition of LXRα activity may require AMPK activation and AMPK-LXRα signaling activation may be VTE’s underlying mechanism against NASH.

Direct antagonism of LXRα may lead to increased cholesterol biosynthesis and suppressed reverse cholesterol transport in the liver (Hong and Tontonoz, 2014). However, an obvious cholesterol-raising action by VTE *in vivo* was not seen (Figure 2D and Supplementary Figure S1). Conversely, VTE lowered hepatic TC levels in DIO mice. AMPK activation can inhibit hepatic cholesterol biosynthesis. Thus, it also hints at indirect inhibition of LXRα by VTE-triggered AMPK activation may avoid adverse hypercholesteremia effects from LXRα antagonism.

In summary, our work suggests that VTE activates AMPK-LXRα signaling *in vitro* and *in vivo*. VTE supplementation ameliorated hepatic steatosis, inflammation, and liver damage in DIO and MCDD-fed mice. VTE decreased liver cell apoptosis and ER stress in NASH. We confirmed the protective effects of vine tea against NASH. VTE gives rise to AMPK activation and suppresses AMPK-mediated LXRα activity. Our findings suggest that VTE may become a novel therapy for NASH.

## Data Availability

The raw data supporting the conclusion of this article will be made available by the authors, without undue reservation.

## Glossary

ABCA1: ATP-binding cassette, sub-family A, member 1
ABCG1: ATP-binding cassette, sub-family G, member 1
ALT: alanine aminotransferase
AMPK: adenosine monophosphate-(AMP)-activated protein kinase
AST: aspartate transaminase
cle-CASP3: cleaved Caspase-3
CPT1: carnitine palmitoyltransferase 1
CYP7A1: cholesterol 7-alpha hydroxy-lase
DIO: diet-induced obesity
DMSO: dimethylsulfoxide
DMY: dihydromyricetin
ER: endoplasmic reticulum
FAS: fatty acid synthase
FXR: farnesoid X receptor
HDL-c: HDL cholesterol
HE: haematoxylin and eosin
HF: high fat
HFD: High-fat diets
HPLC: high-performance liquid chromatography
IL1β: interleukin 1β
IL6: interleukin 6
IR: insulin resistance
LBD: ligand-binding domain
LDL-c: LDL cholesterol
LXR: liver X receptor
MCDD: methionine and choline-deficient l-amino acid diets
MCSD: methionine and choline supplement diet
MTT: thiazolyl blue tetrazolium bromide
NAFL: non-alcoholic fatty liver
NAFLD: non-alcoholic fatty liver disease
NASH: non-alcoholic steatohepatitis
PPAR: peroxisome proliferator-activated receptor
RLUs: relative luciferase units
SCD1: stearoyl-Coenzyme A desaturase 1
SREBP-1c: sterol regulatory element binding transcription factor 1c
TC: total cholesterol
TG: triglyceride
TNFα: tumour necrosis factor α
TUNEL: terminal deoxynucleotidyl transferase dUTP nick end labeling
VTE: vine tea extract
