# Supplementary figures and images for: Extracts of Vine Tea Improve Diet-Induced Non-Alcoholic Steatohepatitis Through AMPK-LXRα Signaling
Source: Front Pharmacol. 2021 Jul 30;12:711763. doi: 10.3389/fphar.2021.711763 (PMC8361841; doi:10.3389/fphar.2021.711763)

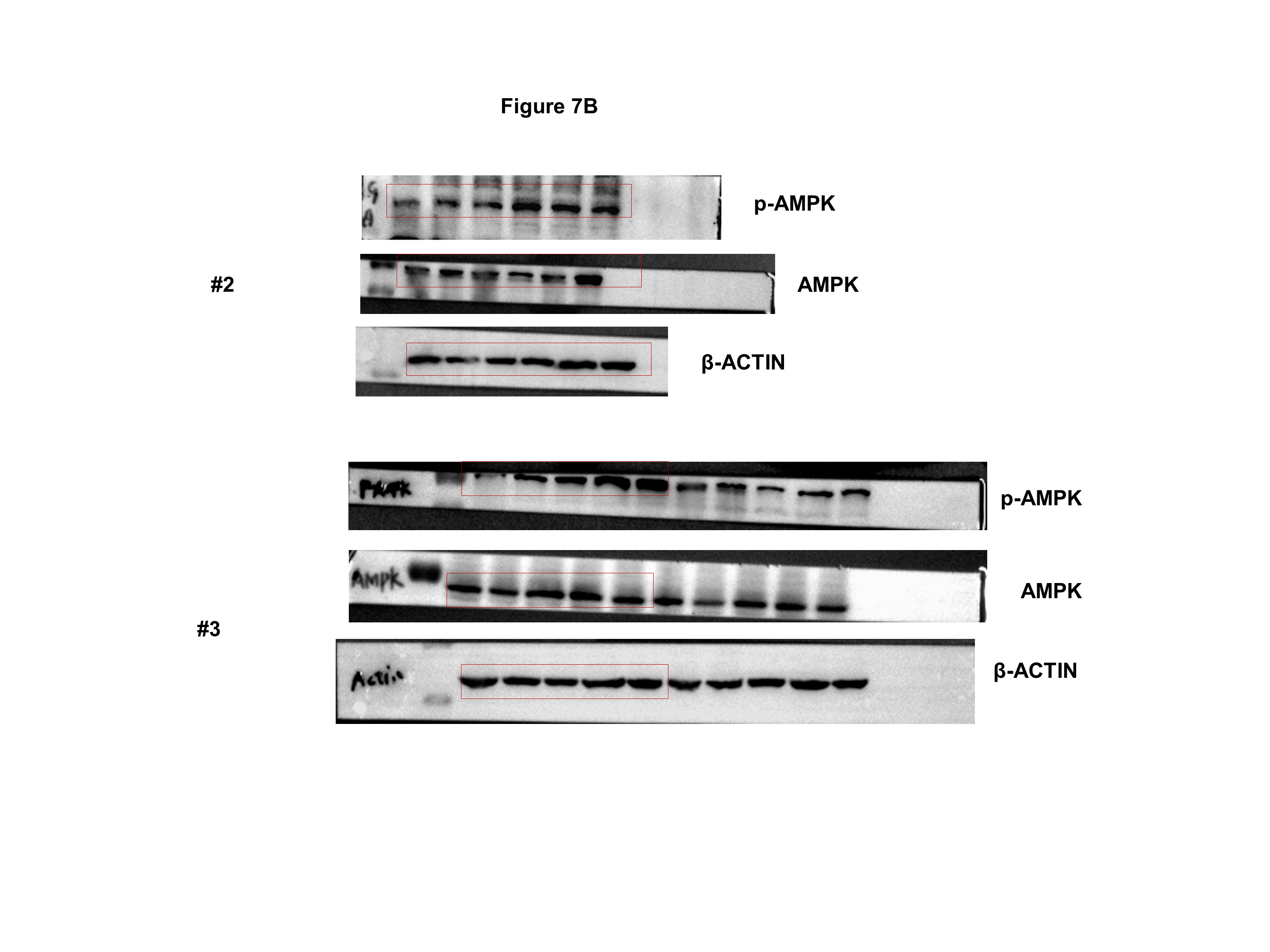

Supplement: Supplementary file 1 [file Image6.TIF]

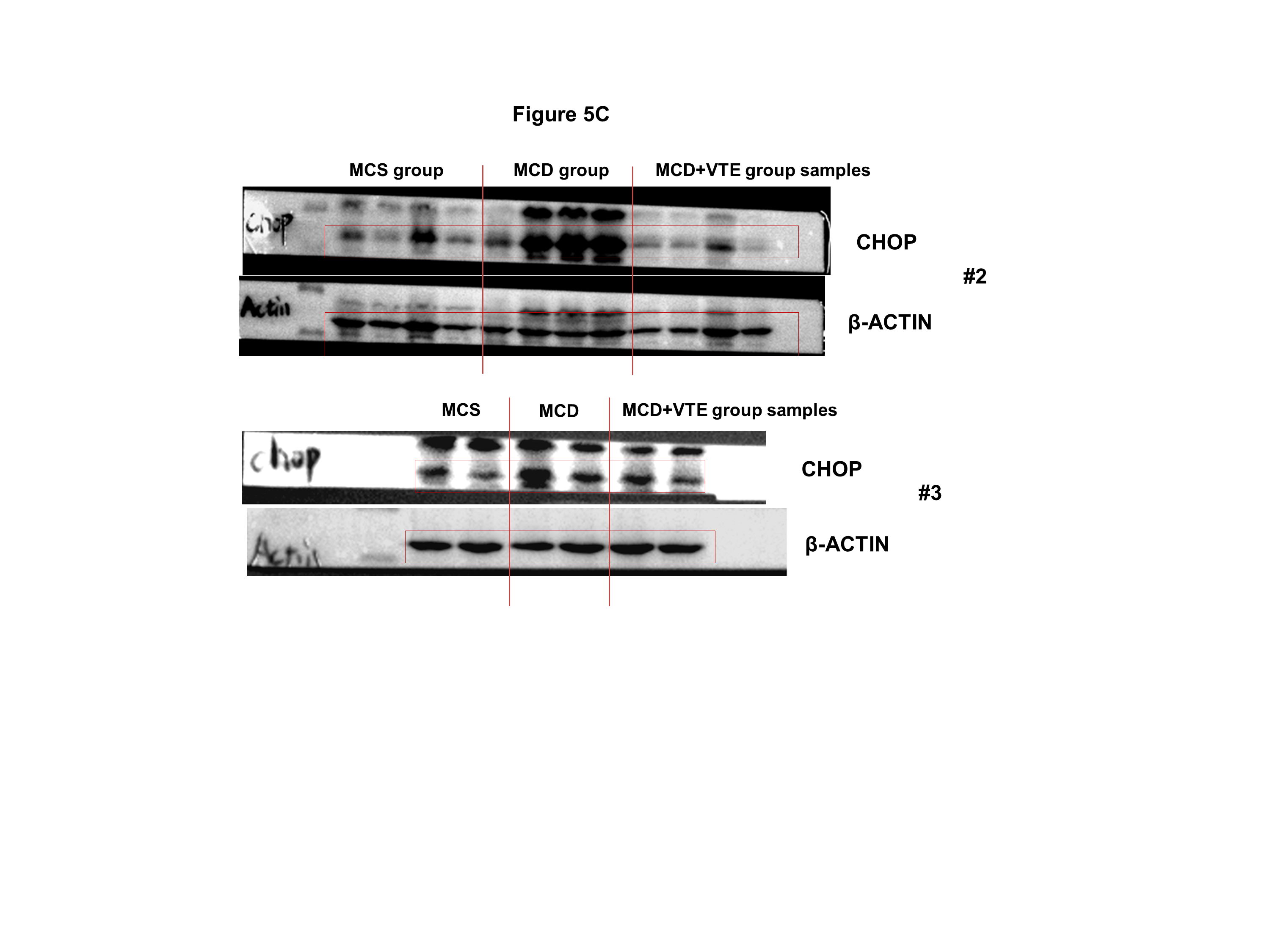

Supplement: Supplementary file 2 [file Image3.TIF]

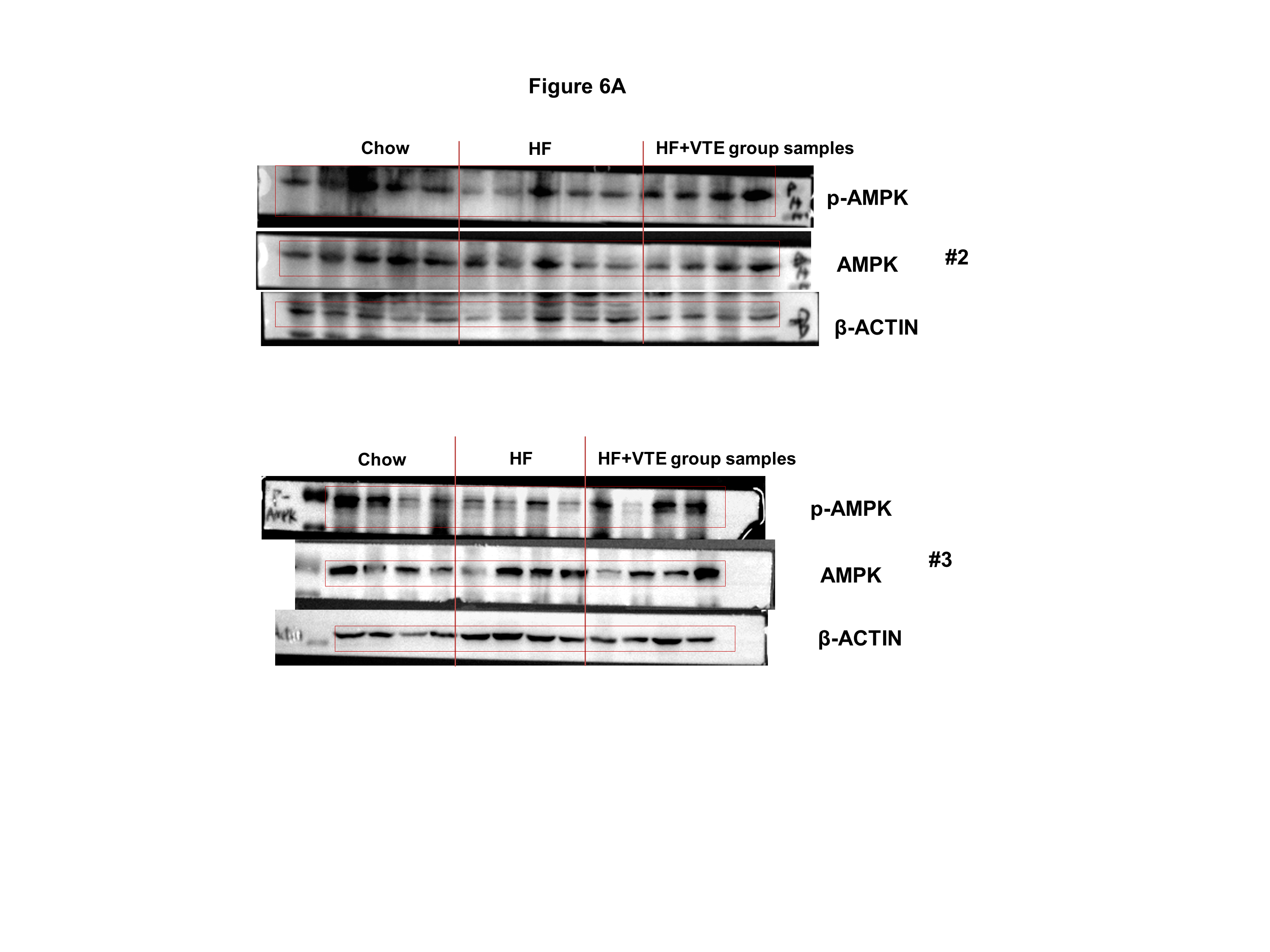

Supplement: Supplementary file 3 [file Image4.TIF]

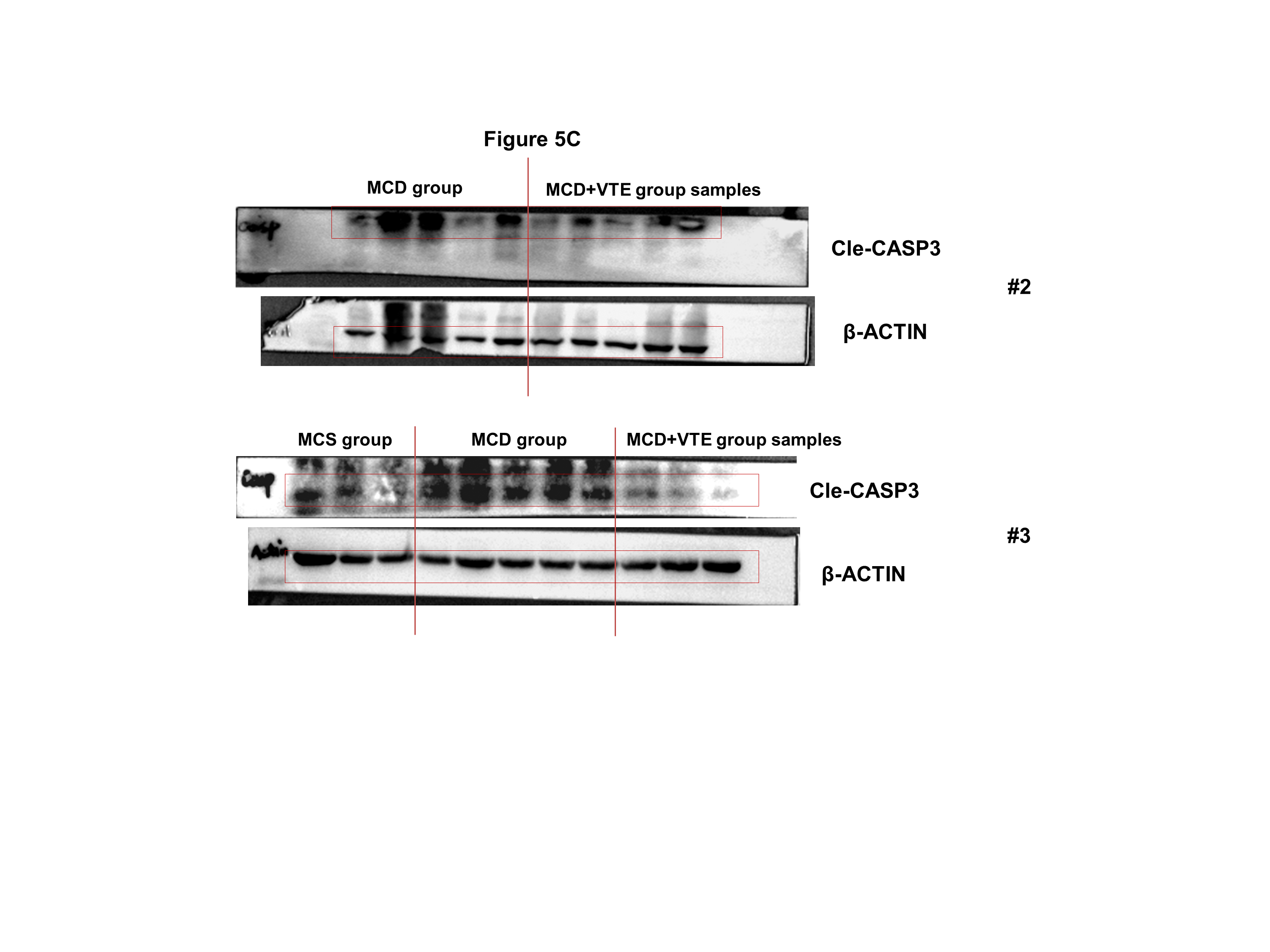

Supplement: Supplementary file 4 [file Image2.TIF]

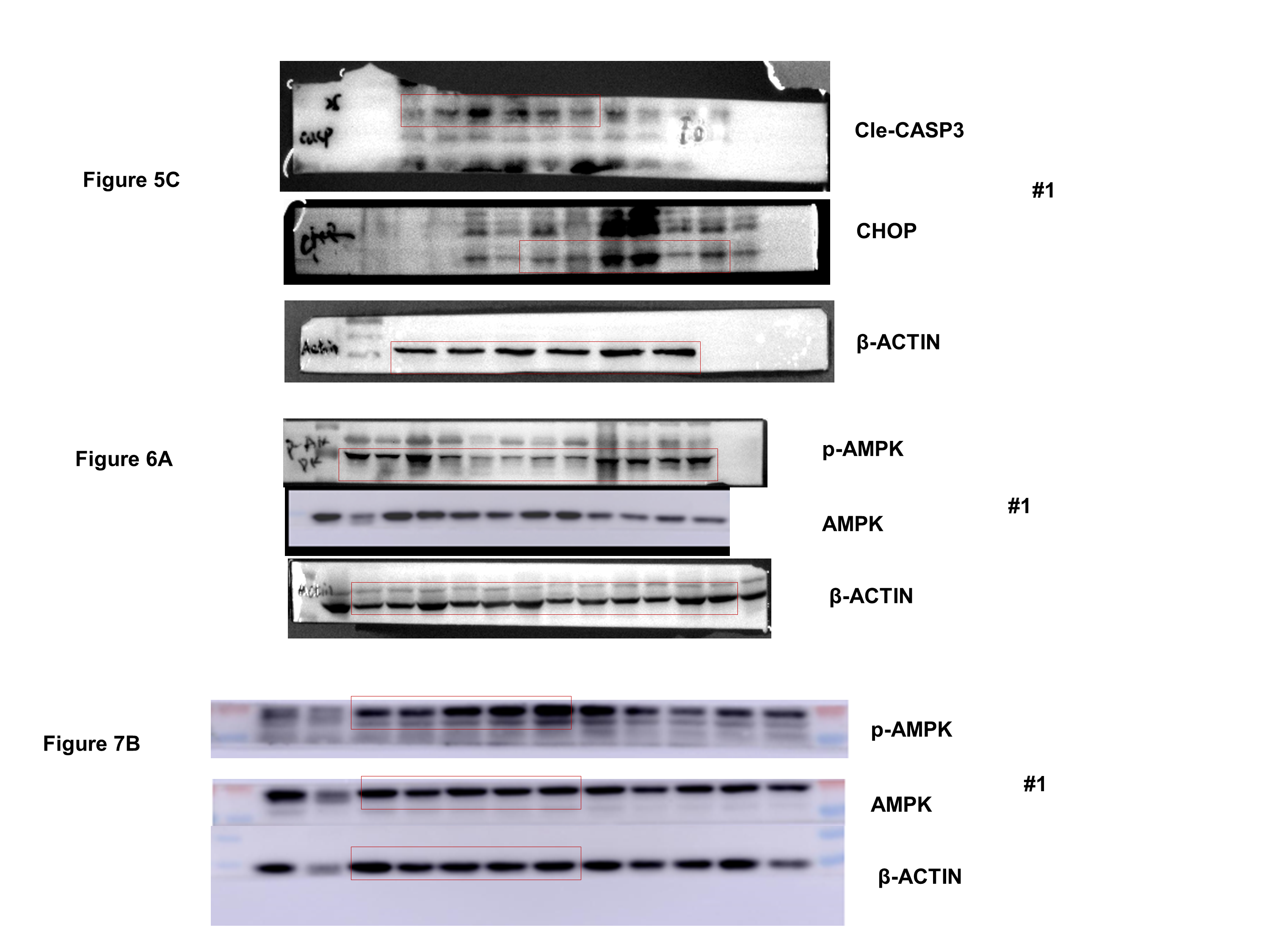

Supplement: Supplementary file 5 [file Image1.TIF]
