# Supplementary material for: Extracts of Vine Tea Improve Diet-Induced Non-Alcoholic Steatohepatitis Through AMPK-LXRα Signaling
Source: Front Pharmacol. 2021 Jul 30;12:711763. doi: 10.3389/fphar.2021.711763 (PMC8361841; doi:10.3389/fphar.2021.711763)
Supplement: Supplementary file 7 [file DataSheet1.docx]

***Supplementary Material***

1. **VTE does not raise serum cholesterol levels in MCDD-fed mice.**

To explore whether VTE affected cholesterol metabolism in mice from MCD treatment, the levels of serum total cholesterol (TC) and LDL cholesterol (LDL-c) in MCSD-, MCDD-, and MCDD+VTE-fed mice were determined. The results indicate that VTE does not increase serum TC and LDL-c levels in MCDD-fed mice (Supplementary Figure 1A and B).


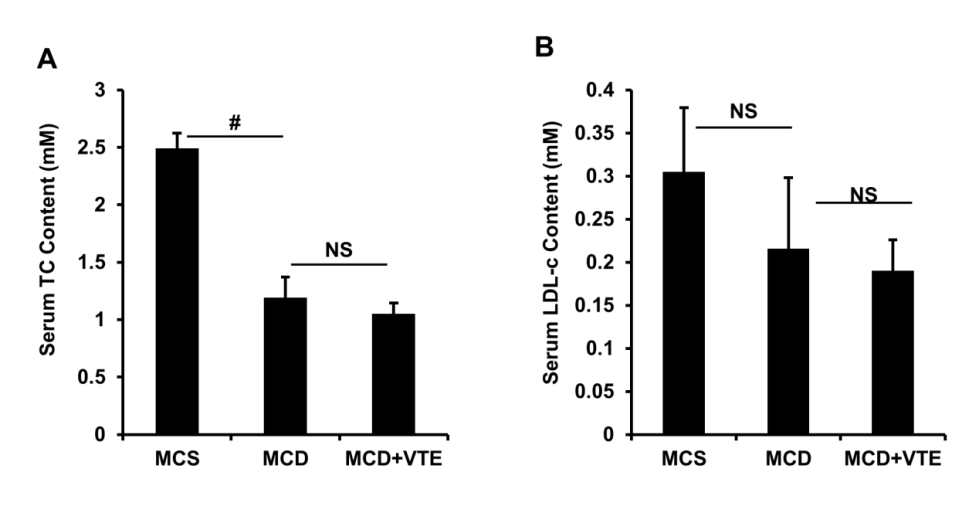


**Supplementary Figure 1. VTE has no effect on serum TC and LDL-c metabolism in MCDD-fed mice.** Male C57BL/6J mice were kept on MCDD in the presence and absence of VTE (1 g.100 g^-1^ diet) for 6 weeks. (A) Serum total cholesterol (TC). (B)low-density lipoprotein cholesterol (LDL-c). Data was statistically analysed as means ± SEM (n = 5-6). ^#^P<0.05, MCD compared with the MCS group. NS, no significance

1. **VTE suppresses LXRα signaling in MCDD-fed mice.**

To study whether VTE could regulate AMPK, LXRα and LXRα target genes expression in MCDD-fed mouse livers, the relative mRNA expression of genes in AMPK-LXRα were determined. As shown in supplementary Figure 2, VTE decreased hepatic mRNA expression of LXRα target genes ABCA1, ABCG1, CYP7A1 and SCD1, but without changing the LXRα and AMPK in MCDD-fed mice (Supplementary Figure 2). Our data indicates VTE down-regulates LXRα signaling in MCDD-fed mouse livers.


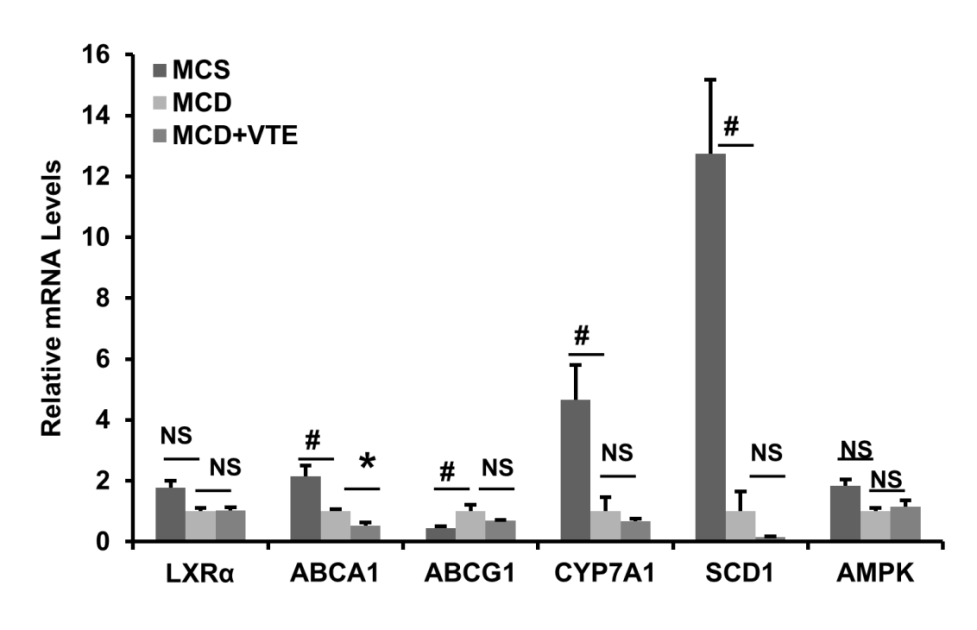


**Supplementary Figure 2. Hepatic LXRα signaling in MCDD-fed mice was inhibited by VTE treatment.** Male C57BL/6J mice were kept on MCDD in the presence and absence of VTE (1 g.100 g^-1^ diet) for 6 weeks. Relative mRNA expression of AMPK and genes for LXRα signaling in MCS, MCD and MCD+VTE group mouse livers were determined. β-ACTIN was used as an internal control for normalizing the mRNA levels. Data are statistically analysed as means ± SEM (n=3 for all groups). ^#^P<0.05, MCS versus MCD group. *P<0.05, MCD versus MCD+VTE group. NS, no significance
